# Preferences for Decision Control among a High-Risk Cohort Offered Lung Cancer Screening: A Brief Report of Secondary Analyses from the Lung Screen Uptake Trial (LSUT)

**DOI:** 10.1177/23814683231163190

**Published:** 2023-03-27

**Authors:** Stefanie Bonfield, Mamta Ruparel, Jo Waller, Jennifer L. Dickson, Samuel M. Janes, Samantha L. Quaife

**Affiliations:** Research Department of Behavioural Science and Health, University College London, London, UK; Lungs for Living Research Centre, UCL Respiratory, University College London, London, UK; School of Cancer and Pharmaceutical Sciences, King’s College London, London, UK; Lungs for Living Research Centre, UCL Respiratory, University College London, London, UK; Lungs for Living Research Centre, UCL Respiratory, University College London, London, UK; Centre for Cancer Prevention, Detection and Diagnosis, Wolfson Institute of Population Health, Barts and The London School of Medicine and Dentistry, Queen Mary University of London, London, UK

**Keywords:** decision control preferences, lung cancer screening, shared decision making

## Abstract

**Background.** Personal autonomy in lung cancer screening is advocated internationally, but health systems diverge in their approach, mandating either shared decision making (with a health care professional) or individual decision making. Studies of other cancer screening programs have found that individual preferences for the level of involvement in screening decisions vary across different sociodemographic groups and that aligning approaches with individual preferences has the potential to improve uptake. **Method.** For the first time, we examined preferences for decision control among a cohort of UK-based high-risk lung cancer screening candidates (*N* = 727). We used descriptive statistics to report the distribution of preferences and chi-square tests to examine associations between decision preferences and sociodemographic variables. **Results.** Most (69.7%) preferred to be involved in the decision with varying degrees of input from a health care professional. Few (10.2%) wanted to make the decision alone. Preferences were also associated with educational attainment. **Conclusion.** These findings suggest one-size-fits-all approaches may be inadequate in meeting diverse preferences, particularly those placing sole onus on the individual.

**Highlights:**

- Preferences for involvement in decision making about lung cancer screening are heterogeneous among high-risk individuals in the United Kingdom and vary by educational attainment.
- Further work is needed to understand how policy makers might implement hybrid approaches to accommodate individual preferences and optimize lung cancer screening program outcomes.

While lung cancer screening facilitates early detection and reduces mortality in high-risk populations, its value depends on the tradeoff between the risk of lung cancer mortality and screening harms, including overdiagnosis and radiation exposure.^1,2^ This tradeoff varies from person to person, meaning that screening decisions are preference sensitive and must take each individual’s values and perspective into account.^3^

However, the ethical ideal of high-quality decision making remains challenging to achieve in practice.^4^ While decisions were once made exclusively by health care professionals, personal autonomy is now mandated internationally through policy and practice guidelines. As part of this process, individuals must therefore be informed about the harms and benefits to contribute to the final decision. This means that health care professionals must receive appropriate training in communicating this information and supporting patient decision making,^5^ although health care systems differ in their approach. In the United States, professional bodies advocate shared decision making (SDM),^6^ in which health care professionals and individuals make the screening decision together.^7^ In the United Kingdom, the National Screening Committee emphasizes personal informed choice based on accurate and accessible screening information, not necessarily involving the opinion of a health care professional.^8^

However, this dichotomy in approach may not accommodate the potentially more diverse preferences of those high-risk adults offered lung cancer screening, as preferences have been found to vary by education in other screening contexts. Studies of colorectal and prostate cancer screening preferences have found that although 45% and 57% of individuals, respectively, preferred to make a shared decision about participating, lower levels of education were associated with greater preference for the health care professional to make all screening decisions.^9,10^ Moreover, a study of lung cancer screening preferences in the United States found that among high-risk individuals of an African American background, only 33.6% preferred to make the screening decision with their doctor.^11^ A hybrid approach may therefore be required that considers individuals’ preferences for the level of involvement they would like in lung cancer screening decisions, which, importantly, has the potential to improve decision quality, screening uptake, and health outcomes.^12,13^

To our knowledge, there are scarce data examining decision control preferences within the eligible lung cancer screening population and no studies within the UK context, where no national lung cancer screening program currently exists. The aim of this study was to explore decision control preferences for lung cancer screening in a sample of high-risk individuals and explore associations between these preferences and sociodemographic characteristics.

## Methods

### Study Design

This study examined decision control preferences among high-risk adults considering lung cancer screening using low-dose computed tomography (LDCT), and explored the distribution of these preferences by sociodemographic characteristics and smoking status. The study methods have been described elsewhere; we report secondary analyses of cross-sectional data collected as part of the Lung Screen Uptake Trial (LSUT),^14^ a randomized controlled demonstration trial primarily aiming to test whether “targeted, stepped and low burden” invitation materials improved screening uptake. These specific analyses were planned after data collection had taken place and were not part of the prespecified statistical analysis plan. Participants provided informed consent, and approval was granted by an NHS Research Ethics Committee (15/LO/1186).

### Participants and Recruitment

Adults aged 60 to 75 y who had been recorded by their primary care physician as having smoked within the past 7 y (*n* = 2,012),were invited to a nurse-led, hospital-based Lung Health Check appointment.

Those who chose to attend the appointment (*n* = 1,005) were asked to report sociodemographic information and their current smoking status and to complete an in-clinic paper-based questionnaire. Decision control preferences were assessed using a single questionnaire item based on the Control Preferences Scale (“In general, who do you think should make the decision about whether you have lung cancer screening?”).^15^ Individuals were asked to select 1 of 5 possible response options. The first option represented health care professional–based decision making, the third and middle options represented SDM, and the fifth option represented individual-based decision making. Thus, the second and fourth options provided a mid-point between health care professional–based decision making and SDM, and SDM and individual-based decision making, respectively (see Table 1).

**Table 1:** Sample Characteristics

	Questionnaire Respondents	Questionnaire Nonrespondents
	*n* = 727	*n* = 273
Gender, *n* (%)		
Male	389 (53.5)	159 (58.2)
Female	338 (46.5)	114 (41.8)
Age, y		
Median	65	66
Min, max	60, 76	60, 76
Ethnicity, *n* (%)		
White	614 (84.5)	212 (77.7)
Asian	9 (1.2)	9 (3.3)
Black	66 (9.1)	34 (12.5)
Mixed	8 (1.1)	3 (1.1)
Other	29 (4.0)	13 (4.8)
Not stated	1 (0.1)	2 (0.7)
Indices of multiple deprivation rank quintile,^a^ *n* (%)		
Quintile 1 (most deprived)	435 (59.8)	166 (60.8)
Quintile 2	266 (36.6)	96 (35.2)
Quintile 3	16 (2.2)	6 (2.2)
Quintile 4	2 (0.3)	0 (0.0)
Missing	8 (1.1)	5 (1.8)
Smoking status,^b^ *n* (%)		
Currently smokes	505 (69.5)	204 (74.7)
Formerly smoked	222(30.5)	69 (25.3)
Education level,^c^ *n* (%)		
Finished school at/before age 15 y^d^	348 (47.9)	177 (64.8)
CSEs, O levels, or equivalent	80 (11.0)	24 (8.8)
A levels, further education (not degree), other or equivalent	119 (16.4)	38 (13.9)
Degree	179 (24.6)	34 (12.5)
Missing	1 (0.1)	0 (0.0)
Relationship status, *n* (%)		
Married/cohabiting	330 (45.4)	97 (35.5)
Single/separated/divorced/widowed	395 (54.5)	176 (64.5)
Prefer not to say	2 (0.3)	0 (0.0)
English as main language, *n* (%)		
Yes	676 (93.0)	228 (83.5)
No^d^	51 (7.0)	45 (16.5)

a The Index of Multiple Deprivation ranks the relative deprivation of every small area in England. This allows post codes to be classified into 5 quintiles, from most to least deprived. No participants in this study lived within the least deprived quintile.

b Those who had never smoked (*n* = 4) and those whose smoking status was unknown (*n* = 1) were excluded.

c In the United States, finishing school at/before 15 would equate to not completing high school, CSEs and A levels would equate to completing high school, and a degree would equate to a 4-year college degree and/or advanced degree.

d Significant difference in proportion of respondents compared with nonrespondents.

Eligibility for LDCT screening was assessed and offered to those eligible and choosing to be screened. All participants were provided with full information about the risks and benefits of screening, which they could discuss with a nurse who supported their comprehension and decision making.

### Statistical Analysis

Data were analyzed in SPSS (version 25). We report descriptive statistics (frequencies and percentages) of sociodemographic variables and decision control preferences. Chi-square tests were used to compare demographic differences between those who completed the questionnaire and those who did not and were also used to explore decision control preferences within sociodemographic variables, including gender, education, ethnicity, first language, smoking status, and relationship status. Pearson’s correlation was used to examine whether decision control preferences varied by age.

### Role of the Funding Source

The funding source was separate from the research team and had no role in the design and conduct of the study; collection, analysis, and interpretation of the data; and preparation, review, or final approval of the manuscript.

## Results

Among the 1,005 patients who attended the health check appointment, 84.5% (*n* = 845) were eligible for an LDCT scan, and among those, 91.2% (*n* = 770) took up the offer (for more information on the LSUT cohort, see Quaife et al., 2020.^14^ The response rate to the in-clinic questionnaire analyzed in the present study was 72.6% (730/1005). Five participants were excluded because they had never smoked or did not provide their smoking status, leaving a group of 727 respondents and 273 nonrespondents.

Compared with nonrespondents (see Table 1), a lower proportion of those who responded to the questionnaire had the lowest level of education (finished school aged ≤15 y: 64.8% v. 47.9%, *P* = 0.001) and spoke a first language other than English (16.5% v. 7.0%, *P* < 0.001).

Decision control preferences among questionnaire respondents were varied. The most frequently reported preference was for the mid-point between SDM and individual-based decision making (“I should make the decision but strongly consider the health professionals opinion”) selected by 30.4%, followed closely by a preference for SDM (27.2%: see Table 2). Meanwhile, the least common preference was for solely individual-based decision making.

**Table 2:** Decision Control Preferences among Questionnaire Respondents

Decision Control Preferences	*n* (%)
Health professionals should make the decision using all that is known about lung cancer screening	146 (20.1)
Health professionals should make the decision but strongly consider my opinion	88 (12.1)
Health professionals and I should make the decision together, on an equal basis (SDM)	198 (27.2)
I should make the decision, but strongly consider the health professional’s opinion	221 (30.4)
I should make the decision, using all I know or learn about lung cancer screening	74 (10.2)

SDM, shared decision making.

**Figure 1:**
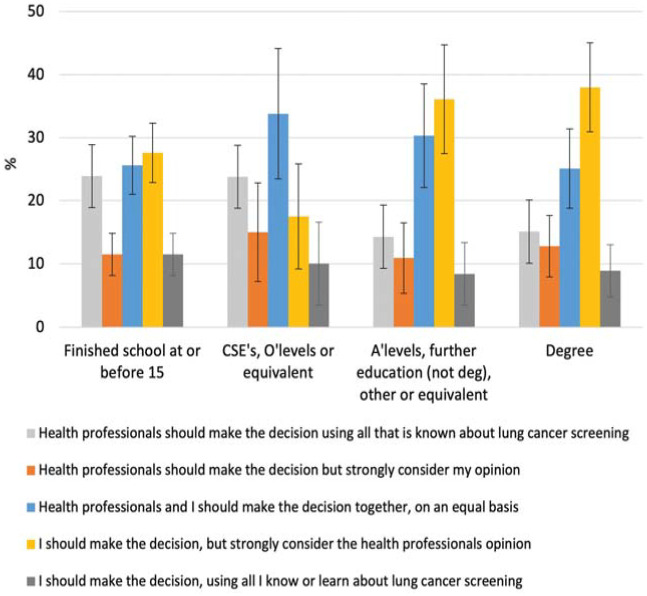
Decision control preferences as a percentage within each education level. Error bars represent 95% confidence intervals.

A 5 × 4 chi-square test revealed a statistically significant association between decision preference and education χ^2^(12) = 21.56, *P* = 0.04. Preferences as a percentage within each education level are shown in Figure 1. Participants in the highest educational category most frequently preferred to make the decision themselves with strong consideration of their health care professionals’ opinion (38.0%). The preferences of those within the lowest educational category were more varied. While the highest proportion also reported a preference for making the decision while strongly considering their health care professionals’ opinion (27.6%), similar numbers reported a preference for SDM (25.6%) or for the health hcare professional to make the decision alone (23.9%).

Preferences were not significantly associated with age, gender, ethnicity, first language, smoking status, or relationship status.

## Discussion

To our knowledge, this study is the first to explore decision control preferences in the United Kingdom lung cancer screening context. Most participants wanted to be involved in the decision alongside the health care professional (69.7%), but preferences for the level of shared involvement were heterogeneous. In support of previous research exploring lung cancer screening decision preferences,^11^ only 27.2% of participants in this study preferred to make the decision on an equal basis, while 12.1% wanted the health care professional to make the final decision and 30.4% wanted to make the final decision themselves. Although this demonstrates the value of personal autonomy, few participants (10.2%) wanted to make the decision alone, underscoring the importance of health care professionals’ involvement and SDM approaches as the dominant choice.

Preferences for decision control varied most among participants with relatively lower levels of education, with similar numbers preferring no involvement, shared involvement, or principal involvement (while considering a health care professionals’ opinion). This heterogeneity in preference by education is particularly important to understand because high-risk candidates for screening are overrepresented among those with lower levels of education, but lower education has been associated with lower screening uptake among individuals who currently smoke.^16^ Therefore, approaches to invitation that demand individual-based decision making prior to attendance may not be equitable.

There are some limitations to interpreting the findings from this study. It should be noted that the analyses reported were exploratory and not guided by directional hypotheses. Moreover, the number of comparative tests conducted warrants planned analyses to replicate and strengthen these findings. The sample cohort was limited to individuals aged between 60 and 75 y who had chosen to attend an in-person lung health check and subsequently showed high levels of LDCT screening uptake (91.2%). It is therefore possible these findings are due to a cohort effect given the limited age range included and of limited generalisability to all those invited to screening. There was variation in the order in which participants completed the questionnaire and were offered LDCT screening, which may have affected decision preferences. In addition, ethnic minority groups were underrepresented, and those who did not complete the questionnaire (nonrespondents) were more likely to have a lower level of education and speak a first language other than English.

Future research should seek to explore preferences in more diverse samples including younger age groups and those less inclined toward screening. This would allow wider insight into associations between preferences and sociodemographic variables among individuals who are eligible for lung cancer screening. It would also be interesting to explore whether preferences reported in this study persist in other medical decision-making contexts among this population.

In conclusion, one-size-fits-all approaches to shared or individual-based decision making may be inadequate in meeting the diverse preferences of the lung screening population. While the US position on SDM aligns with most preferences in this study, a singular approach may overlook the preferences of some individuals in high-risk populations. Further research is needed to understand how individual preferences might be accommodated through hybrid approaches to decision making by lung cancer screening programs.
